# The influence of postural threat-induced anxiety on locomotor learning and updating

**DOI:** 10.1152/jn.00364.2023

**Published:** 2024-02-07

**Authors:** Toby J. Ellmers, Morgan Durkin, Karthigan Sriranganathan, David J. Harris, Adolfo M. Bronstein

**Affiliations:** ^1^Centre for Vestibular Neurology, Department of Brain Sciences, Imperial College London, London, United Kingdom; ^2^Public Health and Sport Sciences, University of Exeter Medical School, Exeter, United Kingdom

**Keywords:** anxiety, emotion, feedforward control, motor adaptation, motor control

## Abstract

The ability to adapt our locomotion in a feedforward (i.e., “predictive”) manner is crucial for safe and efficient walking behavior. Equally important is the ability to quickly deadapt and update behavior that is no longer appropriate for the given context. It has been suggested that anxiety induced via postural threat may play a fundamental role in disrupting such deadaptation. We tested this hypothesis, using the “broken escalator” phenomenon: Fifty-six healthy young adults walked onto a stationary walkway (“BEFORE” condition, 5 trials), then onto a moving walkway akin to an airport travelator (“MOVING” condition, 10 trials), and then again onto the stationary walkway (“AFTER” condition, 5 trials). Participants completed all trials while wearing a virtual reality headset, which was used to induce postural threat-related anxiety (raised clifflike drop at the end of the walkway) during different phases of the paradigm. We found that performing the locomotor adaptation phase in a state of increased threat disrupted subsequent deadaptation during AFTER trials: These participants displayed anticipatory muscular activity as if expecting the platform to move and exhibited inappropriate anticipatory forward trunk movement that persisted during multiple AFTER trials. In contrast, postural threat induced during AFTER trials did not affect behavioral or neurophysiological outcomes. These findings highlight that actions learned in the presence of postural threat-induced anxiety are strengthened, leading to difficulties in deadapting these behaviors when no longer appropriate. Given the associations between anxiety and persistent maladaptive gait behaviors (e.g., “overly cautious” gait, functional gait disorders), the findings have implications for the understanding of such conditions.

**NEW & NOTEWORTHY** Safe and efficient locomotion frequently requires movements to be adapted in a feedforward (i.e., “predictive”) manner. These adaptations are not always correct, and thus inappropriate behavior must be quickly updated. Here we showed that increased threat disrupts this process. We found that locomotor actions learned in the presence of postural threat-induced anxiety are strengthened, subsequently impairing one’s ability to update (or “deadapt”) these actions when they are no longer appropriate for the current context.

## Graphical abstract

**Figure:**
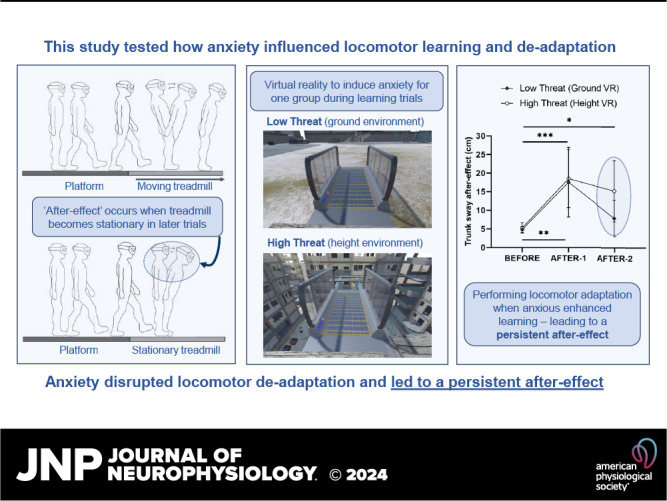

## INTRODUCTION

From stepping over an uneven paving stone to slowing down and stepping more cautiously when approaching a slippery surface, humans must frequently contend with external threats to balance. In such instances, postural stability will be maximized if gait behavior can be adapted in a “feedforward” (i.e., predictive) manner ahead of time (1–3). The effectiveness of any feedforward motor strategy is, however, determined by the accuracy of the prediction itself. If the brain incorrectly predicts the behavior required to contend with the environmental threat, or if the adapted behavior continues after the threat has been navigated, locomotion will be inappropriate for the current context. The continued presence of an adapted motor behavior in a context in which it is no longer appropriate is often referred to as an “aftereffect” (4). Inappropriate feedforward behavior can lead to maladaptive, overly cautious, and potentially destabilizing locomotor adjustments (5–7). It is therefore important that any inappropriate predictive behaviors are quickly updated (i.e., “deadapted”) to avoid the persistence of potentially unsafe locomotor strategies.

Although healthy control subjects are able to use error signals to quickly deadapt inappropriate feedforward behavior (4), this process can be disrupted in clinical populations. An inability to update faulty motor predictions is proposed to underpin a range of maladaptive locomotor behaviors, including “overly cautious” gait in older adults (8) and “higher-order” gait abnormalities such as functional gait symptoms (6, 9). For instance, individuals with functional gait symptoms show an impaired ability to update (or deadapt) feedforward locomotor behaviors that are no longer appropriate for the given context (6). Researchers have proposed that increased anxiety may underpin the persistence of such maladaptive feedforward behavior (6, 8, 10).

This stance is supported by preliminary experimental evidence highlighting how adapting locomotion under postural threat-induced anxiety seems to enhance inappropriate feedforward locomotor behaviors (11). Here, larger aftereffects were observed for individuals who learned to adapt their stepping behavior to a moving surface of faster rather than slower speeds (i.e., greater threat to postural stability). Similar overall patterns of results were reported by Bakkum and Marigold (12). Here, participants who learned to adapt their locomotion under conditions of greater postural threat (misplaced step led to a loss of balance) showed greater generalization of these learned motor patterns. In other words, these participants were more likely to exhibit the learned behavior in different tasks in which the behavior was not appropriate. However, neither study assessed deadaptation rates. This makes it difficult to conclude that increased threat and anxiety underpin the persistence of (i.e., inability to deadapt) inappropriate locomotor behavior, as suggested previously (8, 10).

It is also difficult to directly attribute these previous results specifically to postural threat-induced anxiety. This is because the methods used by both Bakkum and Marigold (12) and Green et al. (11) to induce the postural threat fundamentally changed the physical nature of the task itself, resulting in more substantial losses of balance during the locomotor adaptation phase. This makes it difficult to determine the exact mechanism driving these previous results. For instance, they may be a consequence of the larger error signals experienced rather than the presence of increased threat/anxiety itself. Furthermore, Green et al. (11) observed elevated physiological arousal (i.e., increased perceived threat) throughout both the adaptation (i.e., “learning”) and deadaptation phases. Consequently, the precise mechanism through which anxiety affects locomotor deadaptation (if at all) remains unclear. For example, although anxiety is known to enhance memory consolidation [meaning that locomotor behaviors learned when anxious may be harder to “unlearn” (13, 14)], one alternative explanation may be that anxiety during the deadaptation phase biases behavior toward the learned, familiar response (15). Untangling the direct contribution that postural threat-induced anxiety plays in the maintenance of inappropriate locomotion (i.e., independent from error signal magnitude), and the precise mechanism(s) to address, is crucial for developing effective interventions for persistent inappropriate feedforward locomotor behaviors.

The aim of the present study was therefore to explore how postural threat-induced anxiety, manipulated via a virtual reality (VR) manipulation without altering the physical nature of the task itself [unlike previous studies (11, 12)], influences locomotor adaptation and deadaptation. Specifically, we asked whether increased postural threat-induced anxiety leads to persistent abnormal postural behavior that overrides ascending sensory information and conscious intention. To enhance our understanding of the mechanisms through which anxiety affects locomotor learning and updating, we also aimed to manipulate the specific point at which threat and anxiety are induced: during either the learning (i.e., adaptation phase; *experiment 1*) or deadaptation (*experiment 2*) phase. Based on previous work (11, 12), we hypothesize that postural threat-induced anxiety primarily disrupts locomotor deadaptation by strengthening the initial encoding of the motor memory. We therefore predict that inducing threat during the locomotor learning phase (*experiment 1*) will lead to an enhanced locomotor aftereffect, as well as subsequently slower deadaptation (i.e., persistent locomotor aftereffect). In contrast, we predict that inducing threat and anxiety during the locomotor deadaptation phase (rather than the learning phase; *experiment 2*) will actually blunt the locomotor aftereffect, given the perceived consequences of triggering an inappropriate locomotor strategy and losing one’s balance during this condition.

## METHODS

### Participants

Fifty-six young adults (mean ± SD age = 22.5 ± 4.3 yr; 38 females, 18 males) participated in the research. Twenty-eight of these participants completed *experiment 1* (mean ± SD age = 24.9 ± 4.9 yr; 17 females, 11 males), and 28 participated in *experiment 2* (mean ± SD age = 24.1 ± 3.7 yr; 21 females, 7 males). See Table 1 for further demographic information. Previous research has reported large effect sizes (partial eta squared values of 0.24 and higher) when investigating the effects of increased threat on locomotor deadaptation with a comparable experimental paradigm and outcomes (11). A power calculation conducted with G*Power (v3.1.9.2) showed that 28 participants per experiment would be required to detect a significant within-between interaction of a more conservative medium effect size (*f* = 0.25; assuming 1 − β = 80%, α = 0.05, 2 groups, and 3 within-subject conditions).

**Table 1.** Participant demographics

Variable	*Experiment 1*		*Experiment 2*	
	Control	Threat-Learn	Control	Threat-After
Age, yr	25.9 (5.6)	24.1 (4.3)	25.4 (4.3)	22.8 (2.7)
Sex (females)	7/14	10/14	11/14	10/14
Height, cm	172.5 (8.1)	167.4 (12.0)	168.1 (6.6)	166.5 (5.8)
Weight, kg	70.4 (11.0)	66.6 (12.8)	66.3 (9.1)	66.6 (11.5)
VR experience (yes)	1/14	0/14	3/14	1/14
STAI, Trait (20-80)^a^	32.6 (9.2)	33.0 (8.7)	35.5 (9.6)	31.9 (6.6)

Variable values are means (SD). STAI, Trait, Trait subscale of the Spielberger State and Trait Anxiety Inventory; VR, virtual reality. ^a^Higher scores indicate greater trait anxiety.

All participants were free from any known musculoskeletal, neuro-otological, or visual disease (apart from conventional optic correcting glasses, which were worn underneath the virtual reality headset in the experiment) and did not report any history of balance disorders. The Imperial College Research Ethics Committee (ICREC) approved the study, and each participant provided written informed consent before participating.

### Data Collection

**Figure 1.**
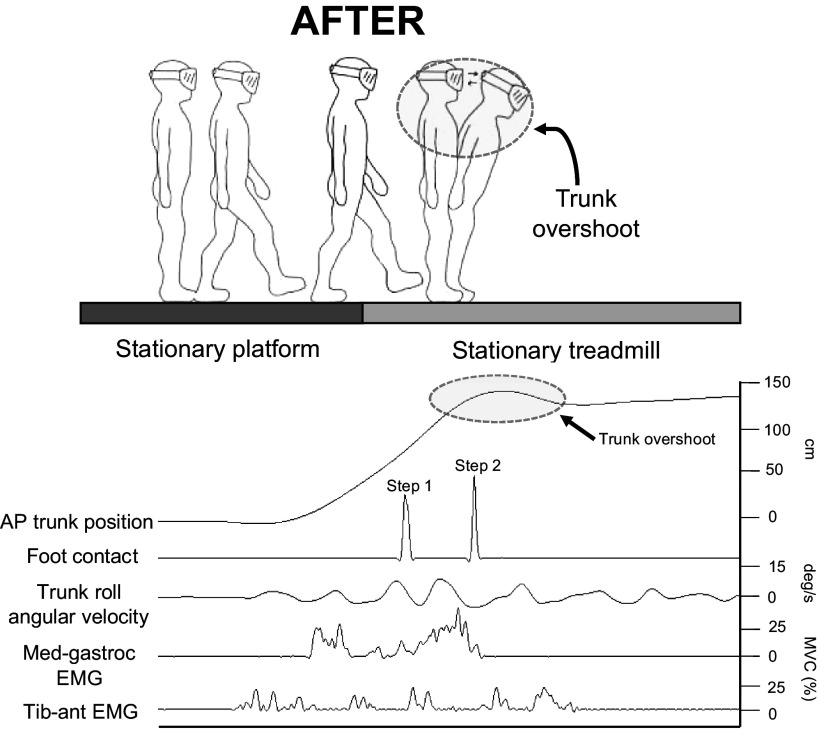
*Top*: schematic diagram of the broken escalator paradigm while wearing the virtual reality headset (AFTER trials pictured; treadmill stationary). Participants initiate gait with their right foot. They then take a second step onto the treadmill with their left foot and a third step to bring the right foot next to the left. The “trunk overshoot” pictured reflects the classic aftereffect in this paradigm, whereby participants retain the adapted rapid trunk movement required to maintain postural stability during MOVING trials. *Bottom*: representative data for the first AFTER trial for a single participant for all kinematic [trunk position in the anterior-posterior (AP) position (used to calculate pre-foot contact gait approach velocity and trunk overshoot) and gyroscope attached to the trunk (used to calculate peak roll angular velocity of the trunk)] and electromyographic (EMG) (anticipatory pre-foot contact and reactive post-foot contact EMG activity of both the medial gastrocnemius and tibialis anterior of the left leg, which steps first onto the treadmill) outcomes. Note that the foot contact variable is assessed via accelerometers placed on the treadmill that detect *z*-axis acceleration following foot contact with the treadmill. MVC, maximal voluntary contraction.

A motorized treadmill was used for this study (HP Cosmos Mercury Med v4.0 LT; Germany). The traveling direction of the belt was reversed so that the treadmill carried participants forward (akin to an airport travelator). Handrails were present at 105 cm high. A solid, stationary platform 142 cm in length was aligned and leveled with the foot of the treadmill. Participants started each trial standing on this solid platform, before stepping onto the treadmill (Fig. 1). The treadmill was controlled by an external computer.

In line with previous research that used an experimental paradigm comparable to that used in the present study (6, 7, 11), sagittal trunk position was measured with a Fastrak electromagnetic linear motion tracking device (Polhemus, VT). The sensor was placed over the C7 vertebrae, along with a gyroscope (CRS43; Silicon Sensing Systems, Plymouth, UK) that was used to calculate general postural instability during the task (peak angular roll velocity of the trunk). Bipolar active surface electrodes (model DE2.1; Delsys Inc, United States) were placed on the medial gastrocnemius (MG) muscle and tibialis anterior (TA) muscles bilaterally, with the left medial malleolus used as a reference. The electromyographic (EMG) signals were amplified and filtered online (20–1,000 Hz; Bagnoli 16; Delsys Inc). Foot-treadmill contact timing was determined by a treadmill-mounted accelerometer (Entran, Watford, UK) and corroborated with an accelerometer attached to the participant’s shank (16). All data were recorded at 2,500 Hz for off-line analysis.

As we used virtual reality (VR) to induce postural threat and anxiety (described below), participants also reported their level of prior experience with VR before participation. To assess general trait levels of anxiety, participants completed the trait version of the Spielberger State Trait Anxiety Inventory [STAI (17)].

### General Paradigm

Participants completed the “broken escalator” paradigm, using a treadmill rather than a linear sled as in early papers (7). The paradigm consisted of three successive conditions: BEFORE, MOVING, and AFTER. Each condition required the participant to step from the stationary platform onto the treadmill when it was either stationary (BEFORE and AFTER conditions) or moving at a constant velocity of 1 m/s (MOVING condition). The start position on the solid platform was altered based on individual preferred step length. This was then marked to ensure within-participant consistency across trials. After an auditory tone, participants took one step forward with the right foot onto the stable platform. They then took a second step onto the treadmill with their left foot and a third step to bring the right foot next to the left (whereby, if moving, the treadmill began to decelerate and then came to a full stop within 500 ms of foot contact). Here they adopted a quiet stance until the recording had finished. The recording for each trial lasted for 15 s.

**Figure 2.**
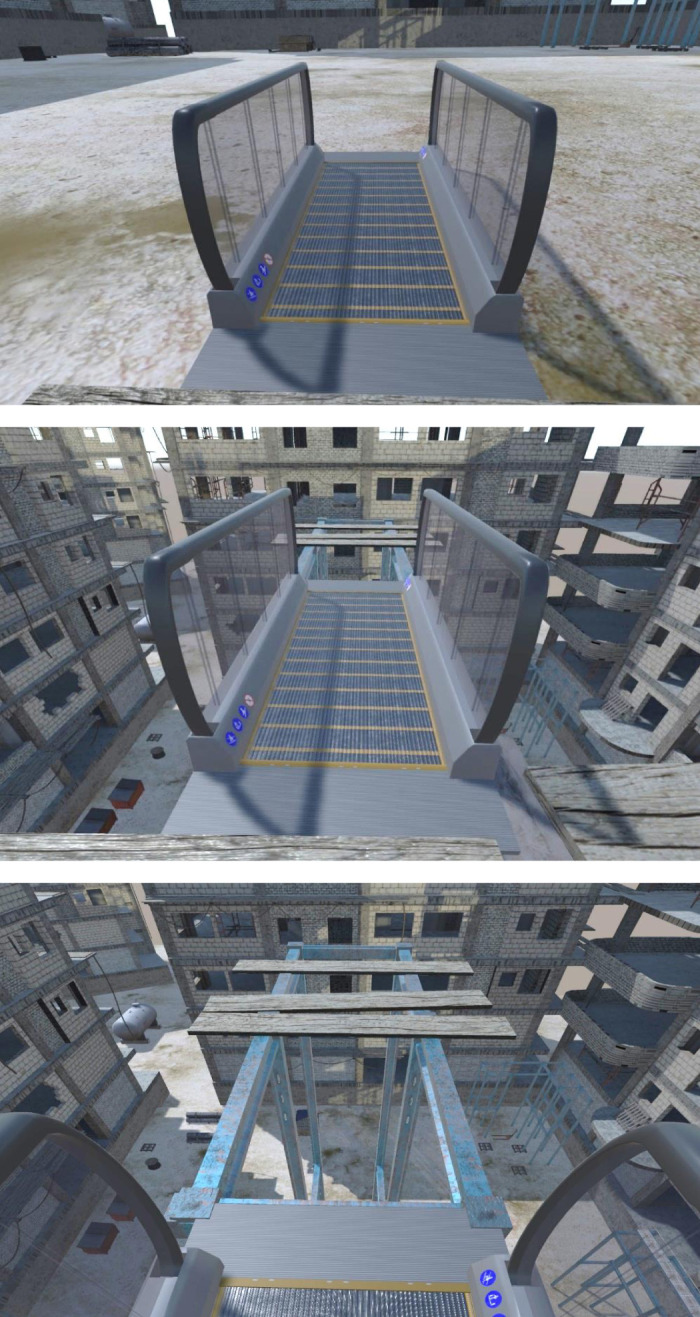
Example of the starting position in the ground-level low-threat (*top*) and elevated high threat (*middle*) virtual reality environment that propelled participants toward a 25-m drop (*bottom*).

Each trial was completed while participants were wearing a wireless HTC Vive VR headset system (HTC Inc., Taoyuan City, Taiwan). The custom VR environment was created with Unity (2019.4; Unity Technologies, California) and featured a stable platform and a travelator that matched the dimensions of the real-world treadmill (Fig. 2). During stationary (BEFORE and AFTER) trials participants saw a stationary travelator, whereas during MOVING trials they saw a travelator that was moving at the same speed at eye level as the real-life treadmill (1 m/s). Interpupillary distance of the headset was altered for each participant before completing any of the trials. Participants then completed a 2-min familiarization period while wearing the headset and viewing a stationary travelator positioned at the low-threat ground level (see below). They were encouraged to step onto the stationary “travelator,” to gain a sense of how the immersive environment matched the real-world treadmill. The familiarization period also allowed us to ensure accuracy of the visual representation of the travelator and to recalibrate the virtual environment or adjust the headset, where necessary.

Participants completed 20 trials in total, split into three separate blocks. All participants first completed five baseline (“BEFORE”) trials in which the treadmill was stationary. They then completed 10 learning trials in which they stepped onto the moving treadmill (“MOVING”). They then completed 5 trials in which the treadmill was once again stationary (“AFTER”). For these trials, participants were informed in clear and unequivocal terms that the treadmill belt would no longer move. Although participants could not see the treadmill belt itself (because of being immersed in the VR environment), because of the loud noise that the treadmill made when it was moving, each participant confirmed that they believed the experimenters’ warning that the treadmill was stationary during BEFORE and AFTER trials. During each trial, participants were instructed to only use the handrails if absolutely necessary (i.e., if they fell). All but two participants grabbed the handrails in the first MOVING trial. By the third MOVING trial, participants no longer grabbed onto the handrails. Participants did not grab onto the handrail in BEFORE or AFTER trials. As per previous studies using this experimental paradigm (6, 7, 18), we did not discard trials in which participants grabbed onto the handrail, given that this is a standard initial response to the paradigm and as these actions occurred outside the epoch in which our data were analyzed (−250 ms to +1,000 ms after foot contact onto the treadmill; see *Data Analysis*).

We adopt LeDoux and Pine’s (19) definition of anxiety in the present work and view anxiety as a subjective emotional state that occurs when the source of potential harm (e.g., losing one’s balance) is uncertain or distal in space or time. To assess the effects of the postural threat manipulation on subjective anxiety, participants completed a visual analog scale that ranged from 0 (“not at all anxious”) to 10 (“the most anxious I have ever felt”) after each trial to rate the level of anxiety that they felt during the preceding trial (20). Higher scores therefore indicate greater state anxiety.

### Postural Threat Manipulation: *Experiment 1*

*Experiment 1* explored the effects of postural threat induced during MOVING (i.e., learning) trials on locomotor learning and deadaptation. Here, 28 young participants (mean ± SD age = 24.9 ± 4.9 yr; 17 females, 11 males) were recruited and randomly divided into two groups: a low-threat “Control” group and a “Threat-Learn” group. Both groups completed stationary (BEFORE and AFTER) trials while immersed in a low-threat ground-level environment (Fig. 2, *top*). The key difference was that whereas the Control group also completed MOVING trials when immersed in this low-threat environment, the Threat-Learn group performed MOVING trials while immersed in a high-threat environment designed to induce anxiety (Fig. 2, *middle* and *bottom*). This environment required participants to step onto an elevated travelator that propelled them toward a 25-m drop. In each trial, the treadmill/travelator stopped moving before this point, and participants came to a stop near the travelator’s edge (Fig. 2, *bottom*). Virtual reality has been used to reliably induce postural threat, leading to robust emotional and behavioral responses comparable to those experienced when exposed to a real (i.e., nonvirtual) postural threat (21–24).

### Postural Threat Manipulation: *Experiment 2*

**Figure 3.**
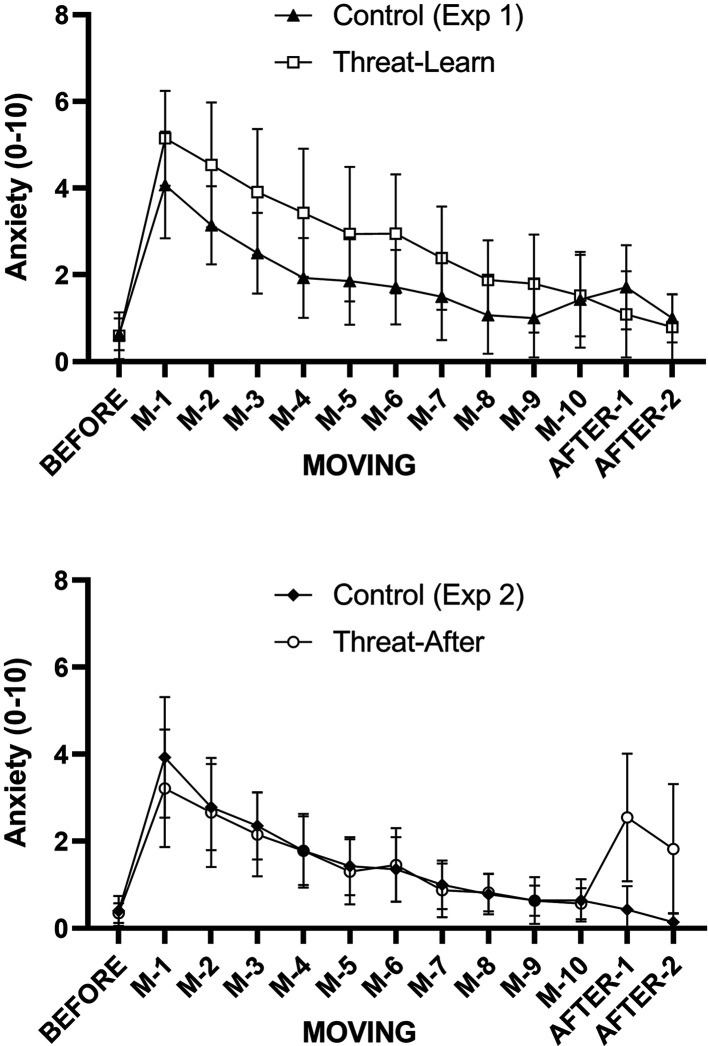
Mean values (and 95% confidence intervals) for self-reported anxiety recorded during BEFORE, MOVING, and AFTER trials during *experiments 1* (*top*) and *2* (*bottom*) (manipulation checks).

We wished to rule out the possibility that the effects observed in *experiment 1* might have been caused by any between-group differences in anxiety observed during AFTER trials (see Fig. 3, *top*, where a trend was observed for Control participants to experience an increase in anxiety during AFTER *trial 1* compared with the Threat-Learn group). *Experiment 2* therefore explored the effects of postural threat induced during AFTER (i.e., deadaptation) trials on locomotor deadaptation. Here, 28 new young participants (mean ± SD age = 24.1 ± 3.7 yr; 21 females, 7 males) were recruited and randomly divided into two groups (“Control” and “Threat-After”).^1^ Both groups completed BEFORE and MOVING trials while immersed in a low-threat ground-level environment (Fig. 2, *top*). Whereas the Control group also completed AFTER trials when immersed in this low-threat environment, the Threat-After group performed AFTER trials while immersed in the high-threat environment described above (Fig. 2, *middle* and *bottom*).

### Data Analysis

#### Kinematic outcomes.

As per previous studies (6, 7, 18), trunk overshoot was calculated from the Fastrak linear trunk position trace. Previous work has shown that participants will counteract the backward destabilization caused by stepping onto the forward-moving surface during MOVING conditions by rapidly moving their trunk forward, with this behavior triggered in a feedforward manner (16). Consequently, increased trunk overshoot (illustrated in Fig. 1, *top*) during AFTER trials is the primary measure of a locomotor aftereffect in this paradigm (5). Trunk overshoot was defined as the maximum forward deviation (“overshoot”) of the trunk, relative to the mean final resting stance position in the last 3 s of the trial, from the Fastrak linear trunk position trace. Because of the nature of the trunk overshoot data, the same or similar criteria could not be used during the MOVING trials; thus trunk overshoot was only calculated for BEFORE and AFTER trials. Direct observation during the experiment showed that seven participants in the Threat-Learn group had a trunk overshoot so large in the first AFTER trial (“AFTER-1”) that they had to take an additional step to avoid falling (a forward stumble). Consequently, trunk overshoot here could not be calculated with the method described above, given that the final resting stance position equated to the overshoot itself. Therefore, for these participants trunk overshoot in AFTER-1 was instead calculated using the difference between the maximum forward deviation of the trunk in AFTER-1 and the mean end position in the last 3 s of the following trial (AFTER-2).

Previous work studying this paradigm has shown that participants will adapt their gait to promote stability during MOVING trials by increasing the velocity at which they approach and step onto the moving treadmill (6, 7, 18). Gait approach velocity was therefore assessed and defined as the mean linear trunk velocity (calculated from the Fastrak linear trunk position trace) in a 250-ms time window before treadmill-foot contact. We used a shorter pre-foot contact epoch compared with previous research to calculate gait approach velocity [which used 500 ms (6, 7, 18)]. This was because we hypothesized that participants in the threatening conditions may be more likely to momentarily pause before stepping onto the treadmill (see Ref. 25), and we did not wish to capture this deceleration and pause in the calculation of gait approach velocity. To provide insight into general postural instability during the task, we also calculated peak angular roll velocity of the trunk from the gyroscope attached to C7. Gyroscope signals were low-pass filtered with a second-order Butterworth filter with a cutoff frequency of 3 Hz (26) and then used to calculate the peak angular roll velocity of the trunk in the 1 s after stepping onto the treadmill. Because of calibration issues, gyroscope data were omitted for one Control participant in both *experiments 1* and *2*.

Trunk overshoot and gait approach velocity were analyzed with in-house customized software (Analysis, D. Buckwell, Medical Research Council) as per previous studies (6, 7, 18), whereas gyroscope data were analyzed with custom-written scripts in MATLAB (The MathWorks, Inc., Natick, MA). Data were assigned a randomized code, and analysis was conducted blinded to group status.

#### EMG outcomes.

EMG data were analyzed in MATLAB (The MathWorks, Inc.) with custom-written scripts. EMG was band-pass filtered at 20–450 Hz, full-wave rectified, and then smoothed using a 50-ms moving average envelope. We then calculated integrated EMG (iEMG) for the MG and TA muscles of the lead stepping leg (i.e., the first leg to step onto the treadmill) in two epochs: 250 ms before foot contact of this leg with the treadmill (i.e., same epoch as gait approach velocity) and 500 ms after foot contact. These epochs represent *anticipatory* and *stabilizing* muscle activity, respectively. iEMG is defined as the sum of the area under the curve divided by the number of data points. EMG signals were normalized to maximal activity induced during an isometric maximal voluntary contraction for each respective muscle (standing on tiptoes on each leg to activate the MG muscles and pulling toes up as hard as possible against isometric resistance to activate the TA muscles). Because of the considerable motion artifacts introduced during MOVING trials, EMG analyses were restricted to BEFORE and AFTER trials [as per previous studies using the broken escalator paradigm (6)]. Data were assigned a randomized code, and analysis was conducted blinded to group status.

### Statistical Analysis

*Experiments 1* and *2* were analyzed separately for all analyses.

We first conducted manipulation checks to confirm the effectiveness of the postural threat manipulation on state anxiety levels. This consisted of using a generalized estimating equation (GEE) [2 group levels (Control vs. Threat-Learn/After) × 10 trial levels (MOVING-1 to MOVING-10), with Holm–Bonferroni-corrected post hoc *t* tests to follow up significant effects. We then conducted a Mann–Whitney *U* test (because of data being nonnormally distributed), comparing Control versus Threat-Learn/After in both AFTER-1 and AFTER-2 (Holm–Bonferroni corrections applied).

Next, we compared groups (Control-Exp1 vs. Threat-Learn; Control-Exp2 vs. Threat-After) on kinematic and EMG outcomes during BEFORE trials, using Mann–Whitney *U* tests (as most outcome variables were nonnormally distributed). Comparisons during MOVING and AFTER trials were analyzed with a GEE. We chose an exchangeable working correlation matrix to define dependency among measurements. We ran a separate GEE for each individual variable. MOVING (i.e., adaptation) and AFTER (i.e., deadaptation or aftereffect) trials were analyzed separately. For MOVING trials, we compared BEFORE to the first and last MOVING trial (MOVING-1 and MOVING-10) across groups, using a GEE [2 group levels (Control vs. Threat-Learn/Threat-After) and 3 trial levels (BEFORE vs. MOVING-1 vs. MOVING-10)]. This allowed us to define the presence of locomotor learning and adaptation. The presence of an aftereffect was determined by analyzing factors Group (2 levels: Controls vs. Threat-Learn/Threat-After) and Trial (3 levels: BEFORE vs. AFTER-1 vs. AFTER-2). A significant difference between BEFORE and AFTER-1 would provide evidence of an initial locomotor aftereffect (5, 7, 27). Previous research using this paradigm has shown a universal rapid deadaptation in healthy young adults following a single AFTER trial (5, 7, 27). A significant difference between BEFORE and AFTER-2 would therefore provide evidence of a persistent or enhanced aftereffect (6, 28). As research has shown that any enhanced aftereffects in this paradigm do not persist beyond AFTER-2 [i.e., aftereffects are abolished from AFTER-3 onward (28)], we therefore chose to limit our analyses to AFTER-1 and AFTER-2. For all GEE analyses, Holm–Bonferroni-corrected post hoc *t* tests followed up significant effects.

### Data Availability

All analyzed data required to replicate the analyses presented here (including individual processed EMG data used for the grand averages in Fig. 6 and Fig. 9) can be accessed via an Open Science Framework repository (https://doi.org/10.17605/OSF.IO/BDU8Q).

## RESULTS

### *Experiment 1*: Effects of Increased Threat during MOVING Adaptation Trials

#### Overview of results.

The present findings provide evidence that increased threat and anxiety during the adaptation phase lead to enhanced feedforward locomotor behaviors. Specifically, we observed an anticipatory “braking” EMG activity in the first AFTER trial for the Threat-Learn group only: The MG muscle (needed to prevent the participant from stumbling forward after stepping onto the treadmill) activated before (i.e., midswing) the foot contacted the treadmill. We also observed a persistent aftereffect (i.e., continuing into AFTER-2) for the Threat-Learn group in one of the key kinematic variables in this paradigm: trunk overshoot. No such persistence was observed for the Control group, suggesting that performing a locomotion adaptation task in the presence of elevated anxiety disrupts the subsequent motor deadaptation. These results are discussed in detail below.

#### Manipulation checks.

Self-reported anxiety did not significantly differ between the Control and Threat-Learn groups during BEFORE trials (*Z* = −1.12, *P* = 0.285). There was a main effect of Group (χ^2^ = 4.64, *P* = 0.031) and no significant interaction effect (χ^2^ = 8.18, *P* = 0.517), with anxiety being significantly higher in the Threat-Learn group throughout the MOVING trials. This confirms the success of the anxiety manipulation (Fig. 3, *top*). There was also a significant main effect of Trial number (χ^2^ = 118.87, *P* < 0.001) during MOVING trials, with anxiety tending to decrease between successive trials until *trial 8* (*P* between 0.001–0.073), whereby no further significant reductions were observed (*P* = 1.00). Although there was a tendency for Control participants to feel more anxious during AFTER trials (particularly AFTER-1), self-reported anxiety did not significantly differ between groups in either AFTER-1 (*Z* = −1.57, *P* = 0.137) or AFTER-2 (*Z* = −1.24, *P* = 0.265).

#### BEFORE trials.

There were no significant between-group differences for any kinematic variable (trunk overshoot, *Z* = −0.14, *P* = 0.910; peak trunk angular roll velocity, *Z* = −1.60, *P* = 0.116; gait approach velocity, *Z* = −1.10, *P* = 0.285) or EMG activity in the stepping leg MG (250 ms pre-foot contact, *Z* = −0.14, *P* = 0.890; 500 ms post-foot contact, *Z* = −0.74, *P* = 0.482). EMG activity in the stepping leg TA did, however, tend to be slightly higher for the Control group compared with the Threat-Learn group (250 ms pre-foot contact, *Z* = −2.16, *P* = 0.031; 500 ms post-foot contact, *Z* = −1.70, *P* = 0.094).

#### MOVING trials.

**Figure 4.**
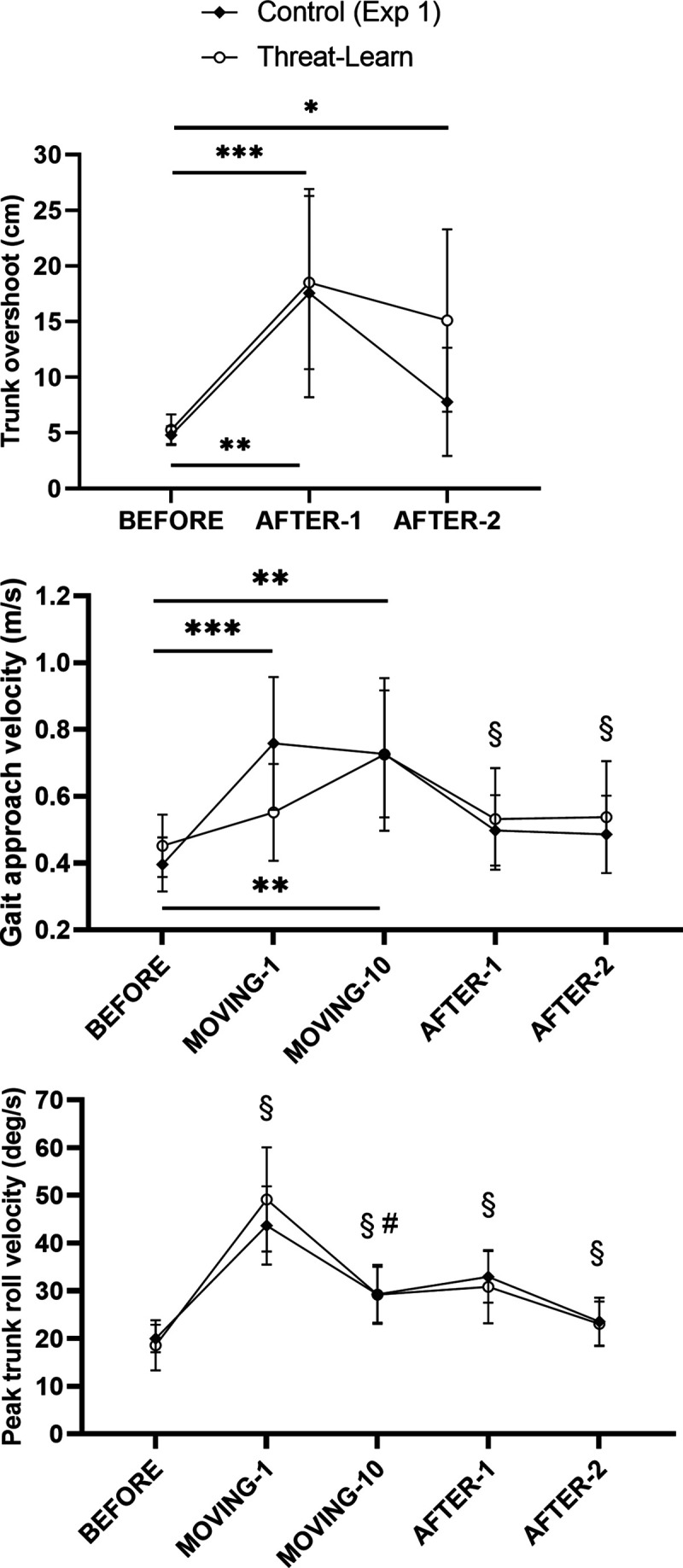
Mean values (and 95% confidence intervals) for kinematic outcomes recorded during BEFORE, MOVING (gait approach velocity and peak trunk roll velocity only), and AFTER trials during *experiment 1*. §Significantly different from BEFORE for both groups (main effect of trial, no interaction); #significantly different from MOVING-1 for both groups (main effect of trial, no interaction); *interaction post hoc test *P* < 0.05; **interaction post hoc test *P* < 0.01; ***interaction post hoc test *P* < 0.001.

As noted in methods, because of the large amounts of EMG movement artifacts introduced when stepping onto the moving treadmill, we only present kinematic data (peak trunk angular roll velocity and gait approach velocity) for MOVING trials (Fig. 3). With respect to peak trunk angular roll velocity, there was a significant main effect of Trial (χ^2^ = 68.09, *P* < 0.001) but neither a significant main effect of Group (χ^2^ = 0.31, *P* = 0.576) nor an interaction effect (χ^2^ = 1.02, *P* = 0.600). This indicates comparable rates of adaptation across groups. For both, there was a substantial increase in postural instability (large increase in peak trunk angular roll velocity) after stepping onto the treadmill during the first MOVING trial, as indicated by a large increase in peak trunk angular roll velocity compared with BEFORE (*P* < 0.001). Peak trunk angular roll velocity significantly decreased in MOVING-10 (compared with MOVING-1; *P* < 0.001), indicating adaptation, although it nonetheless remained elevated compared with BEFORE (*P* < 0.001; Fig. 4, *bottom*).

To maximize postural stability when stepping onto the moving treadmill, one must also increase the speed with which one steps onto the treadmill. Although there was no main effect of Group with respect to gait approach velocity (χ^2^ = 0.39, *P* = 0.533), there was both a significant main effect of Trial (χ^2^ = 38.69, *P* < 0.001) and a significant interaction effect (χ^2^ = 7.36, *P* = 0.025). Post hoc tests revealed that whereas the Control group increased their gait approach velocity in MOVING-1 compared with BEFORE (*P* < 0.001), no such increase was observed in the Threat-Learn group (*P* = 0.284). However, by MOVING-10, the Threat-Learn group had learned to significantly increase their gait approach velocity (compared with BEFORE; *P* = 0.004), with gait approach velocity in MOVING-10 similarly increased (compared with BEFORE) in the Control group as well (*P* = 0.001; Fig. 4, *middle*).

#### AFTER deadaptation trials: overall patterns of results.

**Figure 5.**
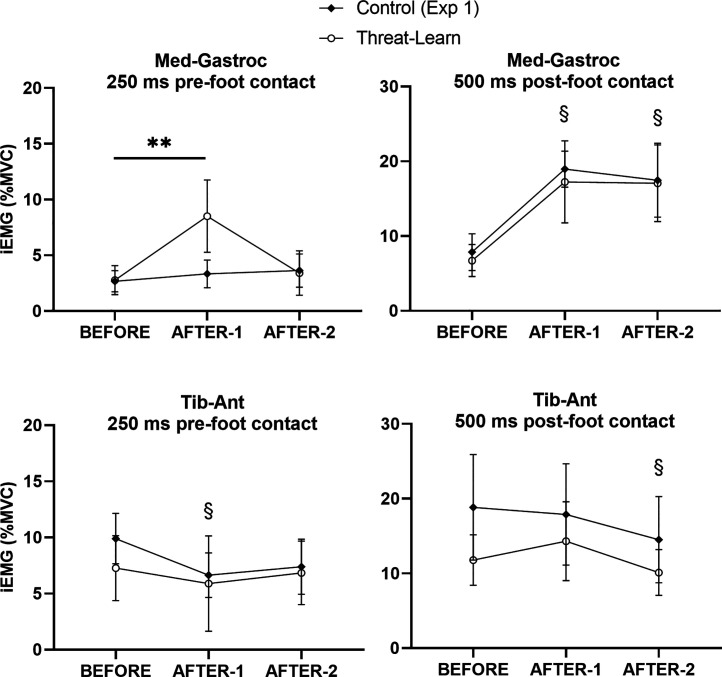
Mean values (and 95% confidence intervals) for electromyographic (EMG) outcomes recorded during BEFORE and AFTER trials during *experiment 1*: stepping leg EMG for medial gastrocnemius and tibialis anterior muscles in an epoch 250 ms before stepping onto the treadmill and 500 ms afterward. iEMG, integrated EMG; MVC, maximal voluntary contraction. §Significantly different from BEFORE for both groups (main effect of trial, no interaction); **interaction post hoc test *P* < 0.01.

**Figure 6.**
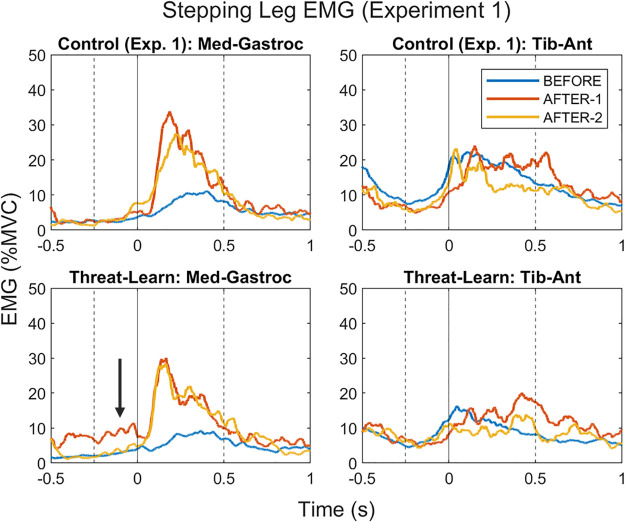
Electromyographic (EMG) grand averages for stepping leg medial gastrocnemius and tibialis anterior muscles [presented as % of maximal voluntary contraction (MVC)] during *experiment 1*. The solid line at *time point 0* reflects foot contact of the stepping leg onto the treadmill. The dashed lines at −250 ms and +500 ms reflect the pre (i.e., anticipatory)- and post (i.e., reactive)-foot contact epochs during which EMG outcomes were analyzed. Note the arrow highlighting the anticipatory EMG activity during the first AFTER trial (AFTER-1) for the medial gastrocnemius that was present for the Threat-Learn group only.

In general, the pattern of results for the AFTER trials match those presented previously in healthy control subjects (e.g., Refs. 7, 18, 27, 29): There was a large initial aftereffect for all groups, with respect to kinematic (trunk overshoot and peak angular roll velocity of the trunk and gait approach velocity) and post-foot contact EMG outcomes during the first AFTER trial. As illustrated in Fig. 5 (and the grand averages presented in Fig. 6), the key between-group difference during the first AFTER trial related to additional anticipatory (i.e., in the 250 ms before foot contact) braking EMG activity for the Threat-Learn group only. This anticipatory behavior is illustrated by the arrow in the EMG grand averages in Fig. 6. Trunk movements largely returned to BEFORE levels by the second AFTER trial, whereas gait approach velocity and post-foot contact EMG activity tended to remain elevated for all groups. However, the Threat-Learn group continued to exhibit a large and persistent aftereffect for trunk overshoot, viewed as a key aftereffect behavior in this paradigm (5), during AFTER-2 (Fig. 4, *top*). These results are presented in detail below.

#### AFTER trials: kinematic outcomes.

Although there was no main effect of Group with respect to trunk overshoot (χ^2^ = 0.86, *P* = 0.355), there was both a significant main effect of Trial (χ^2^ = 26.81, *P* < 0.001) and a significant interaction effect (χ^2^ = 7.56, *P* = 0.023). Subsequent post hoc tests revealed an increase in trunk overshoot from BEFORE to AFTER-1 in both groups (Control, *P* = 0.010; Threat-Learn, *P* < 0.001). Trunk overshoot significantly decreased in AFTER-2 compared with AFTER-1 for both groups (Control, *P* = 0.006; Threat-Learn, *P* < 0.001), although this decrease was substantially smaller for the Threat-Learn group (mean decrease = 3.41 cm) compared with the Control group (mean decrease = 9.77 cm). Consequently, trunk overshoot remained significantly elevated in the second AFTER trial for the Threat-Learn group only (*P* = 0.028; Control, *P* = 1.00). This indicates a large and persistent aftereffect.

Evidence of aftereffects into the second AFTER trial was observed across both groups with respect to peak trunk angular roll velocity after treadmill-foot contact as well as gait approach velocity. For peak trunk angular roll velocity, there was a significant main effect of Trial (χ^2^ = 54.02, *P* < 0.001) but neither a significant main effect of Group (χ^2^ = 0.22, *P* = 0.637) nor an interaction effect (χ^2^ = 0.25, *P* = 0.885). Peak trunk angular roll velocity increased in AFTER-1 compared with BEFORE (*P* < 0.001). It then decreased in AFTER-2 (compared with AFTER-1; *P* < 0.001) but still remained elevated compared with BEFORE (*P* = 0.002). Similar results were observed for gait approach velocity. There was a significant main effect of Trial (χ^2^ = 10.24, *P* = 0.006) but neither a significant main effect of Group (χ^2^ = 0.49, *P* = 0.486) nor an interaction effect (χ^2^ = 0.16, *P* = 0.922). Gait approach velocity increased in AFTER-1 (compared with BEFORE; *P* = 0.006) and remained elevated in AFTER-2 (compared with BEFORE; *P* = 0.040).

#### AFTER trials: EMG outcomes.

There was evidence of an enhanced aftereffect, with respect to pre-foot contact EMG activity of the stepping leg MG muscle, for the Threat-Learn group. There was a significant main effect of Trial (χ^2^ = 16.01, *P* < 0.001) and Group (χ^2^ = 5.08, *P* = 0.024); however, the significant interaction effect (χ^2^ = 10.42, *P* = 0.005) and the subsequent post hoc tests revealed that this was driven by a significant increase in EMG activity in AFTER-1 compared with BEFORE for the Threat-Learn group only (*P* = 0.002; Control group, *P* = 0.820). This aftereffect reflecting anticipatory braking muscle activity did not, however, persist into AFTER-2 (*P* = 1.00). In contrast, the significant main effect of Trial (χ^2^ = 9.12, *P* = 0.010) for stepping leg TA activity [but no main effect of Group (χ^2^ = 0.74, *P* = 0.389) or interaction effect (χ^2^ = 2.23, *P* = 0.329)] revealed a significant decrease in pre-foot contact EMG activity in the stepping leg TA in AFTER-1 compared with BEFORE (*P* = 0.012) across groups. EMG activity increased back to BEFORE levels in AFTER-2 (*P* = 0.148).

EMG activity of the stepping leg after foot contact with the treadmill revealed comparable patterns across groups. There was a significant main effect of Trial (χ^2^ = 88.99, *P* < 0.001) but neither a significant main effect of Group (χ^2^ = 0.28, *P* = 0.595) nor an interaction effect (χ^2^ = 0.23, *P* = 0.892) with respect to the MG. EMG activity increased in AFTER-1 (compared with BEFORE; *P* < 0.001) and remained elevated in AFTER-2 (compared with BEFORE; *P* < 0.001). With respect to post-foot contact EMG activity in the stepping leg TA; there was a significant main effect of Trial (χ^2^ = 12.35, *P* = 0.002) but neither a significant main effect of Group (χ^2^ = 2.95, *P* = 0.086) nor an interaction effect (χ^2^ = 0.99, *P* = 0.611). There was no significant difference in EMG activity between BEFORE and AFTER-1 (*P* = 0.683), but EMG activity significantly decreased in AFTER-2 (compared with BEFORE, *P* = 0.012). EMG grand averages for *experiment 1* are presented in Fig. 6.

### *Experiment 2*: Effects of Increased Threat during AFTER Deadaptation Trials

#### Overview of results.

Recall that *experiment 2* was conducted to confirm that the results observed during *experiment 1* were a direct consequence of increased threat and anxiety during the learning phase (i.e., MOVING trials) rather than any differences experienced during AFTER deadaptation trials. In contrast to the Threat-Learn group in *experiment 1*, we did not observe any evidence of enhanced (e.g., anticipatory EMG activity) or persistent (e.g., substantial trunk overshoot in AFTER-2) feedforward locomotor behaviors during the deadaptation phase in *experiment 2*. Instead, the pattern of results for both groups in *experiment 2* matched the Control group from *experiment 1*, as well as those presented previously in healthy control subjects in other studies (e.g., Refs. 7, 18, 27, 29).

In summary, there was a large initial aftereffect for both groups, with respect to kinematic and post-foot contact EMG outcomes. Whereas EMG activity tended to remain elevated in the second AFTER trial [as per previous studies (7, 18)], kinematic aftereffects decreased to levels observed in previous broken escalator work (5, 7, 18, 30), although they remained slightly elevated compared with BEFORE. These results are discussed in detail below.

#### Manipulation checks.

Self-reported anxiety did not significantly differ between the Control and Threat-After groups during BEFORE trials (*Z* = −0.60, *p* = 0.571). During MOVING trials, there was similarly no main effect of Group (χ^2^ = 1.03, *P* = 0.310) or significant interaction effect (χ^2^ = 8.79, *P* = 0.457), indicating that both groups responded to the (low threat) MOVING condition with similar levels of anxiety. The significant main effect of Trial number (χ^2^ = 92.45, *P* < 0.001) revealed that anxiety significantly decreased between successive trials (all *P* < 0.038) until MOVING *trial 7*, whereby no further significant decreases were observed (all *P* > 0.197). Anxiety was significantly higher in the Threat-After group compared with Control during both AFTER-1 (*Z* = −3.42, *P* = 0.001) and AFTER-2 (*Z* = −3.48, *P* = 0.001), once again confirming the success of the anxiety manipulation (Fig. 3, *bottom*).

#### BEFORE trials.

There were no significant between-group differences for any of the kinematic (trunk overshoot, *Z* = −1.52, *P* = 0.137; peak trunk angular roll velocity, *Z* = −0.73, *P* = 0.488; gait approach velocity, *Z* = −0.05, *P* = 0.982) or stepping leg EMG (MG activity 250 ms pre-foot contact, *Z* = −1.29, *P* = 0.198; TA activity 250 ms pre-foot contact, *Z* = −0.46, *P* = 0.963; MG activity 500 ms post-foot contact, *Z* = −0.09, *P* = 0.946; TA activity 500 ms post-foot contact, *Z* = −0.14, *P* = 0.910) variables.

#### MOVING trials.

**Figure 7.**
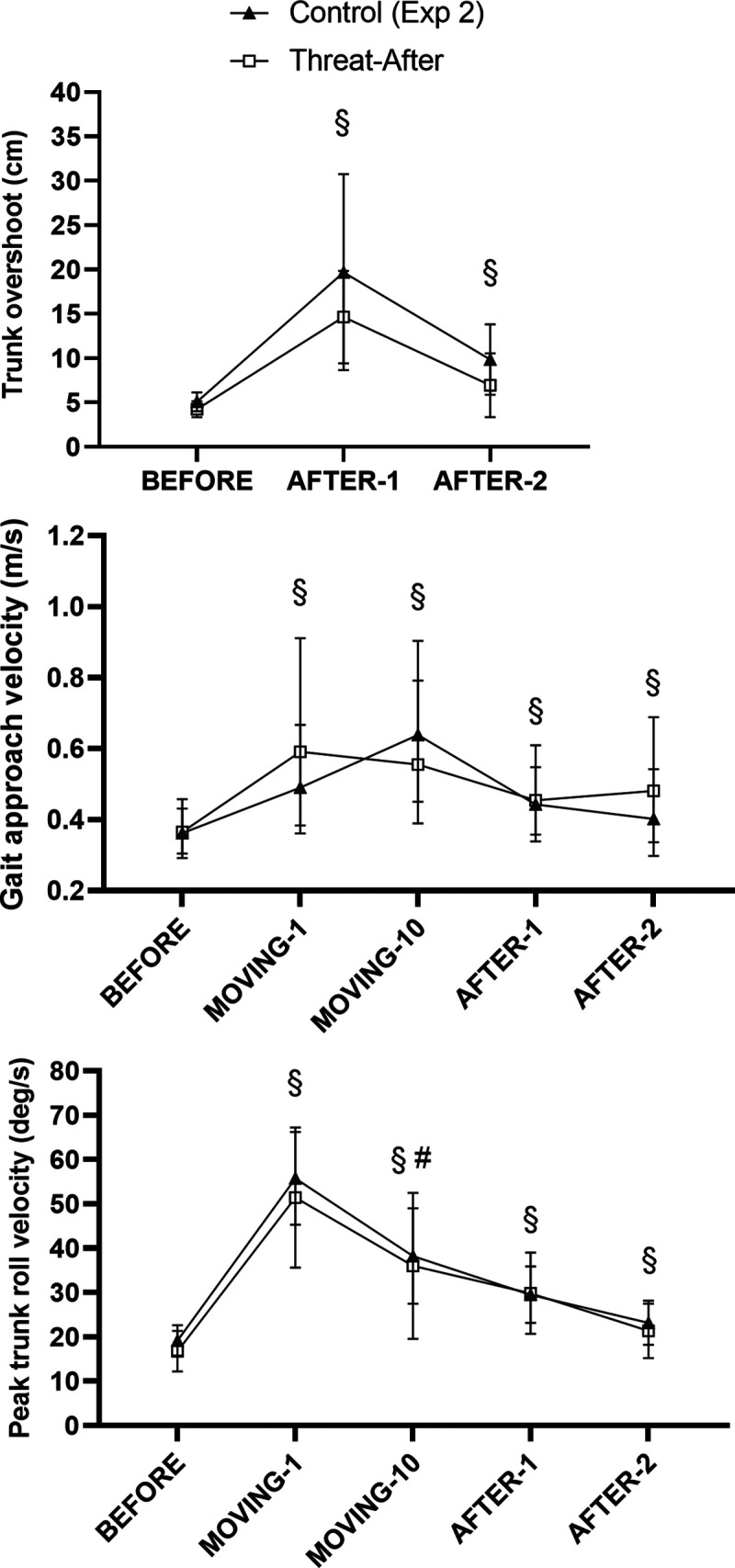
Mean values (and 95% confidence intervals) for kinematic outcomes recorded during BEFORE, MOVING (gait approach velocity and peak trunk roll velocity only), and AFTER trials during *experiment 2*. §Significantly different from BEFORE for both groups (main effect of trial, no interaction); #significantly different from MOVING-1 for both groups (main effect of trial, no interaction).

There was a significant main effect of Trial (χ^2^ = 86.22, *P* < 0.001) but neither a significant main effect of Group (χ^2^ = 0.05, *P* = 0.824) nor an interaction effect (χ^2^ = 0.12, *P* = 0.943) for peak trunk angular roll velocity. This indicates comparable rates of adaptation for the two groups. As with *experiment 1*, peak angular roll velocity of the trunk in the 1 s after stepping onto the treadmill significantly increased from BEFORE to MOVING-1 (*P* < 0.001). It then significantly decreased in MOVING-10 (compared with MOVING-1; *P* < 0.001), although it remained elevated compared with BEFORE (*P* < 0.001). Likewise, for gait approach velocity there was a significant main effect of Trial (χ^2^ = 37.70, *P* < 0.001) but neither a significant main effect of Group (χ^2^ = 0.19, *P* = 0.667) nor an interaction effect (χ^2^ = 1.85, *P* = 0.397). Unlike with the Threat-Learn group in *experiment 1*, gait approach velocity significantly increased in MOVING-1 (compared with BEFORE; *P* = 0.001) and remained significantly elevated in MOVING-10 compared with BEFORE (*P* < 0.001) for both groups. These data are presented in Fig. 7, *middle* and *bottom*.

#### AFTER deadaptation trials: overall patterns of results.

**Figure 8.**
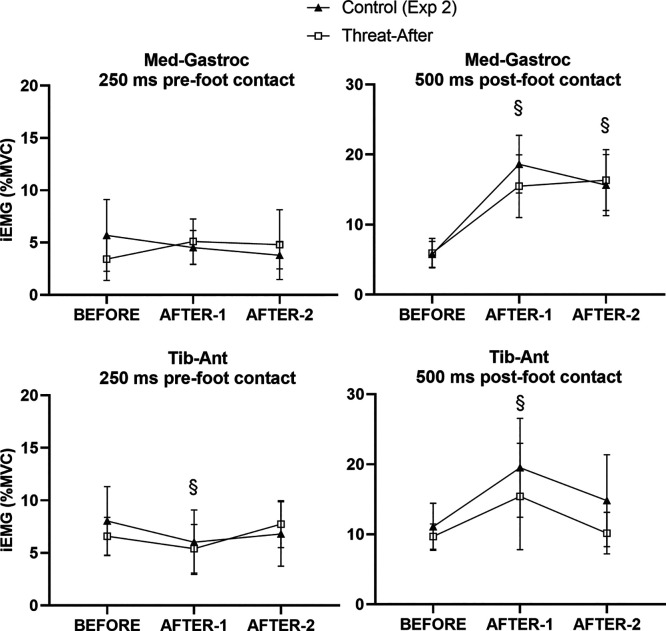
Mean values (and 95% confidence intervals) for electromyographic (EMG) outcomes recorded during BEFORE and AFTER trials during *experiment 2*: stepping leg EMG for medial gastrocnemius and tibialis anterior muscles in an epoch 250 ms before stepping onto the treadmill and 500 ms afterward. iEMG, integrated EMG; MVC, maximal voluntary contraction. §Significantly different from BEFORE for both groups (main effect of trial, no interaction).

In general, the pattern of results for the AFTER trials in *experiment 2* was comparable across the Control and Threat-After groups (kinematic outcomes: Fig. 7; EMG outcomes: Fig. 8) and largely matched those observed for the Control group in *experiment 1*. There was a large initial aftereffect for all groups, with respect to kinematic (trunk overshoot and peak angular roll velocity of the trunk and gait approach velocity) and post-foot contact EMG outcomes during the first AFTER trial, which then decreased in AFTER-2 to levels comparable to those reported in the second AFTER trial in previous research (7, 18, 29). These results are presented in detail below.

#### AFTER trials: kinematic outcomes.

Although the mean aftereffect for trunk overshoot tended to be larger for the Control group in *experiment 2*, there was no significant main effect of Group (χ^2^ = 1.47, *P* = 0.226) or significant interaction effect (χ^2^ = 1.05, *P* = 0.591) and only a significant main effect of Trial (χ^2^ = 23.88, *P* < 0.001) for this variable. As with both groups in *experiment 1*, a large aftereffect was observed during AFTER-1 (*P* < 0.001; Fig. 7, *top*). This aftereffect significantly decreased in AFTER-2 (compared with AFTER-1; *P* < 0.001). Although trunk overshoot remained significantly elevated in AFTER-2 compared with BEFORE (*P* = 0.001), the aftereffect observed here (3.74 cm) was considerably smaller than the mean aftereffect observed during AFTER-2 for the Threat-Learn group in *experiment 1* (9.81 cm) and was instead comparable to the mean aftereffect for the Control group in *experiment 1* (2.99 cm).

A pattern of results was observed in *experiment 2* comparable to that in *experiment 1* with respect to peak trunk roll angular velocity: There was a significant main effect of Trial (χ^2^ = 45.37, *P* < 0.001) and neither a significant main effect of Group (χ^2^ = 0.01, *P* = 0.919) nor a significant interaction effect (χ^2^ = 1.56, *P* = 0.459). As with *experiment 1*, peak trunk angular roll velocity increased in AFTER-1 compared with BEFORE (*P* < 0.001). It then decreased in AFTER-2 (compared with AFTER-1; *P* = 0.004) but still remained elevated in AFTER-2 compared with BEFORE (*P* = 0.003; Fig. 7, *bottom*).

Similar patterns of results were observed for gait approach velocity. There was a significant main effect of Trial (χ^2^ = 17.82, *P* < 0.001) and neither a significant main effect of Group (χ^2^ = 0.79, *P* = 0.375) nor a significant interaction effect (χ^2^ = 2.30, *P* = 0.317). As with *experiment 1*, gait approach velocity significantly increased in AFTER-1 (compared with BEFORE; *P* < 0.01) and remained elevated in AFTER-2 (compared with BEFORE; *P* = 0.002; Fig. 7, *middle*).

#### AFTER trials: EMG outcomes.

Overall, EMG activity was comparable between groups during AFTER trials in *experiment 2*. In contrast to the pre-foot contact anticipatory braking EMG activity observed for the Threat-Learn group in *experiment 1*, there was neither a significant main effect of Group (χ^2^ = 0.04, *P* = 0.841) or Trial (χ^2^ = 0.71, *P* = 0.701) nor an interaction effect (χ^2^ = 2.41, *P* = 0.300) with respect to pre-foot contact EMG activity in the stepping leg MG. As with *experiment 1*, the significant main effect of Trial (χ^2^ = 15.98, *P* < 0.001) for pre-foot contact TA activity [but no main effect of Group (χ^2^ = 0.06, *P* = 0.811) or interaction effect (χ^2^ = 4.66, *P* = 0.097)] revealed a significant decrease in EMG activity in AFTER-1 (*P* < 0.001), which returned to BEFORE levels by AFTER-2 (*P* = 0.947).

**Figure 9.**
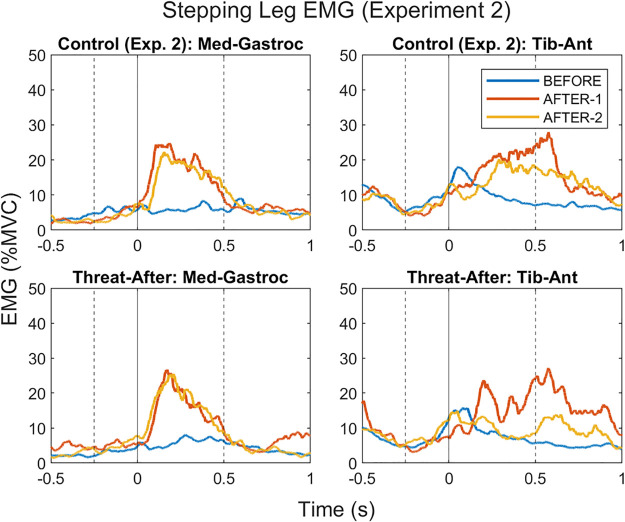
Electromyographic (EMG) grand averages for stepping leg medial gastrocnemius and tibialis anterior muscles [presented as % of maximal voluntary contraction (MVC)] during *experiment 2*. The solid line at *time point 0* reflects foot contact of the stepping leg onto the treadmill. The dashed lines at −250 ms and +500 ms reflect the pre (i.e., anticipatory)- and post (i.e., reactive)-foot contact epochs during which EMG outcomes were analyzed. Note the lack of anticipatory pre-foot contact medial gastrocnemius activity that was present for the Threat-Learn group in *experiment 1*.

With respect to post-foot contact EMG, there was a significant main effect of Trial (χ^2^ = 72.38, *P* < 0.001) but neither a significant main effect of Group (χ^2^ = 0.16, *P* = 0.690) nor an interaction effect (χ^2^ = 4.70, *P* = 0.095) for the stepping leg MG muscle. As with *experiment 1*, EMG activity increased in AFTER-1 (compared with BEFORE; *P* < 0.001) and remained elevated in AFTER-2 (compared with BEFORE; *P* < 0.001). With respect to post-foot contact EMG activity in the stepping leg TA; there was a significant main effect of Trial (χ^2^ = 12.41, *P* = 0.002) but neither a significant main effect of Group (χ^2^ = 1.45, *P* = 0.228) nor an interaction effect (χ^2^ = 1.73, *P* = 0.421). EMG activity in the stepping leg TA increased in AFTER-1 (compared with BEFORE, *P* = 0.002). It then significantly decreased in AFTER-2 (compared with AFTER-1, *P* = 0.004), resulting in a statistically nonsignificant difference between AFTER-2 and BEFORE (*P* = 0.090). EMG grand averages for *experiment 2* are presented in Fig. 9.

## DISCUSSION

This study explored how postural threat-induced anxiety, experimentally induced during either the adaptation or deadaptation phase of the broken escalator paradigm, affects feedforward locomotor behavior. Previous work exploring the role of anxiety in locomotor adaptation induced postural threat in a manner that fundamentally altered the dynamics of task performance, resulting in greater performance errors (11, 12). It is therefore difficult to isolate the specific role of increased postural threat-induced anxiety from the increased error signals experienced. Furthermore, participants in this previous work tended to remain anxious beyond the threat-inducing “learning” trials (11). This makes it difficult to identify the mechanisms through which postural threat-induced anxiety alters locomotor learning and deadaptation.

We built on this existing work by using a novel VR manipulation to increase the perceived threat and induce anxiety, without altering the dynamics of the task being performed. We also manipulated threat and anxiety during distinct and isolated aspects of the task. Our findings revealed that postural threat-related anxiety induced during the adaptation phase (MOVING trials) led to enhanced inappropriate locomotor behaviors that persisted once the threatening context was removed (i.e., prolonged aftereffect in EMG and kinematic outcomes). In contrast, postural threat and anxiety induced during the deadaptation phase (AFTER trials) did not result in altered behavior compared with the Control (low threat) group. These novel findings reveal that motor actions learned in the presence of heightened perceived threat and anxiety are strengthened, leading to difficulties in using motor error signals to deadapt maladaptive behavior (i.e., an enhanced and persistent aftereffect). They also provide direct evidence that it is the presence of threat and anxiety during motor learning, rather than during motor deadaptation, that disrupts the normal processes through which inappropriate motor actions are typically updated.

In the present study, the Threat-Learn group displayed anticipatory EMG activity in the medial gastrocnemius muscle during the first stationary “after” trial (AFTER-1), as if expecting the stationary treadmill to move. Recall that braking muscular activity of the medial gastrocnemius is required to stop participants from falling forward once they step onto the treadmill. This anticipatory EMG activity was not present in any of the other three groups (Control-Exp1, Control-Exp2, Threat-After), nor has it been observed previously when healthy control subjects completed the paradigm (7, 18). Furthermore, as no changes in the TA muscle were observed during this same epoch in this trial for the Threat-Learn group, this rules out that these results were merely a consequence of a generalized increase in muscular activity (i.e., a cocontraction response). Likewise, as (reactive) EMG activity in the medial gastrocnemius following foot contact during AFTER trials did not differ between conditions, this similarly rules out the possibility that this finding is the consequence of general increases in tonic muscle activity. Instead, it implies that this result reflects an aftereffect specific to anticipatory braking muscular activity, suggesting that neuromuscular components of learned locomotor actions are particularly susceptible to the influence of enhanced threat and anxiety.

Although all groups in the present study exhibited an initial aftereffect with respect to trunk overshoot [viewed as a key aftereffect behavior in this paradigm (5)], a prominent aftereffect persisted into the second AFTER trial for the Threat-Learn group (mean trunk overshoot of ∼15 cm vs. ∼5 cm in BEFORE trials; see Fig. 4). Although there was a tendency for trunk overshoot to remain slightly elevated in AFTER-2 for the other groups (Control-Exp1, Control-Exp2, Threat-After), these values were in line with the aftereffects observed in AFTER-2 during previous broken escalator experiments [∼3–4 cm (11, 18)]. Overall, our findings reveal that increased postural threat-induced anxiety during locomotor adaptation leads to individuals being less able to “let go” of these learned behaviors when the threatening environment ceases. The present findings support the recent proposal made by Bakkum and Marigold (12) that the presence of a postural threat enhances (loco)motor learning through a “strengthening of synaptic connections in relevant sensorimotor areas where memory of the learned mapping was marked for consolidation” (Ref. 12, p. 11).

It is well accepted that emotionally salient events enjoy enhanced memory consolidation (31), a process that is strongly modulated by the amygdala (13, 14). Interestingly, Jung et al. (32) have recently reported the existence of a novel disynaptic circuit between the amygdala and the cerebellum, an area of the brain that is crucial for effective locomotor adaptation [and subsequent aftereffect exhibition (33–35)]. Subsequent work has also found increased connection strength between the amygdala and the cerebellum corresponding to enhanced emotional memories (36). We therefore propose that increased anxiety may enhance motor adaptation, leading to prolonged aftereffects, via direct modulation of cerebellar activity. This stance is supported by research that describes how increased postural threat and subsequent anxiety result in larger cortical error signals [a key factor driving cerebellar-based motor adaptation (4)] to both self-generated (37) and external (38) postural perturbations. Future work could look to directly test this hypothesis by using functional MRI (fMRI) to examine changes in the strength of amygdala-cerebellar connections during conditions of emotionally salient motor adaptation.

It is important to also note that moderate levels of anxiety were also experienced by the “low”-threat Control groups during the adaptation phase (average anxiety scores of ∼4/10 during the first MOVING trial). This reflects the extent to which the broken escalator paradigm threatens postural stability, with frequent and substantial losses of balance observed during the early phase of adaptation. Perhaps this enhanced threat and anxiety is the reason why the broken escalator paradigm produces such a rapid, large, and consistent aftereffect. Indeed, unlike “less” threatening locomotor adaptation paradigms (e.g., split-belt treadmill tasks) that take many hundreds of trials to produce adaptation and subsequent aftereffects (4, 39), the broken escalator paradigm has been shown to produce an aftereffect following a single adaptation (i.e., MOVING) trial (18). This idea is further supported by research that describes how increasing the threat to balance during split-belt treadmill adaptation (increased speed discrepancy between the 2 treadmill belts) led to faster readaptation when subjects were subsequently reexposed to the split-belt perturbation (39), further reinforcing the role that postural threat-induced anxiety plays with respect to locomotor adaptation.

The present findings have direct implications for the clinical management of persistent, maladaptive feedforward locomotor behavior [e.g., overly cautious gait in older adults, functional gait disorders, persistent/“functional” dizziness (6, 8, 10, 40, 41)]. Previous work has described how such behaviors appear comparatively resistant to error signals that would typically be used to update the faulty motor action (6). Researchers have proposed enhanced threat processing and anxiety to play a role in this disruption (6, 8, 10). The present findings support this stance and provide further insight into the specific role of postural threat-induced anxiety. Specifically, the findings imply that inappropriate feedforward balance behaviors may become entrenched because of the presence of increased perceived threat and anxiety during the initial presentation of the symptom or behavior. This makes it difficult to then update these actions when they no longer serve an adaptive purpose. Rehabilitation may therefore benefit from providing the patient with a safe and nonthreatening environment in which to relearn their movements using, perhaps, a motor learning strategy that relies less on using error signals to update faulty motor actions [e.g., reinforcement learning (42)].

The present findings also have important implications for balance training and fall-prevention physical therapy more broadly. It has previously been reported that performing a locomotor adaptation task in the presence of a postural threat results in the enhanced recall of the learned locomotor behavior up to a week later (12). Based on these findings, Bakkum and Marigold (12) proposed the intriguing notion that rehabilitation outcomes during balance training may be enhanced if the therapist is able to (safely) increase postural threat during task performance. Our present findings provide further support for this idea. We have recently reported how the simple act of constraining a participant’s arm movements during balance tasks (e.g., arms crossed over the chest) increases perceived threat and anxiety (43). This may be an easy way for therapists to safely induce a postural threat during rehabilitation without altering the dynamics of the task being performed, which may lead to enhanced rehabilitation outcomes. Future work should look to systematically test this idea.

In summary, here we determined that the presence of postural threat-induced anxiety during locomotor adaptation leads to enhanced and persistent locomotor aftereffects, with respect to both neurophysiological (EMG) and kinematic outcomes. These findings build on previous work (11, 12) and have implications for the clinical management of persistent, maladaptive feedforward locomotor behaviors [e.g., overly cautious gait in older adults, functional gait disorders (8, 10)]. Future work should seek to directly translate these findings to clinical populations and determine the specific role that postural threat-induced anxiety plays in the development and maintenance of maladaptive locomotor behaviors.

## DATA AVAILABILITY

Data are available at https://doi.org/10.17605/OSF.IO/BDU8Q.

## GRANTS

T. J. Ellmers is supported by a Wellcome Trust Sir Henry Wellcome Fellowship (222747/Z/21/Z). D. J. Harris is supported by a Leverhulme Trust Early Career Fellowship.

## DISCLOSURES

No conflicts of interest, financial or otherwise, are declared by the authors.

## AUTHOR CONTRIBUTIONS

T.J.E., D.J.H., and A.M.B. conceived and designed research; T.J.E., M.D., and K.S. performed experiments; T.J.E., M.D., and K.S. analyzed data; T.J.E. and A.M.B. interpreted results of experiments; T.J.E. prepared figures; T.J.E. drafted manuscript; A.M.B. edited and revised manuscript; T.J.E., M.D., K.S., D.J.H., and A.M.B. approved final version of manuscript.

## Footnotes

^1^We excluded one participant from the Threat-After group because they experienced maximal levels of anxiety during the (low threat) MOVING condition (scores of 10/10), which would have made it difficult to determine whether any differences observed in this participant were due to heightened anxiety during learning or deadaptation. This participant was excluded immediately after data collection (i.e., before any analysis) and is therefore not included in the 14 Threat-After participants presented here.
